# Label-Scarce Ultrasound Biopsy Needle Identification Using Dynamics-Guided Prior Modeling

**DOI:** 10.3390/diagnostics16203427

**Published:** 2026-10-21

**Authors:** Jihyeon Jeon, Hyunseung Ryu, Myeongjin Lee, Eun Hui Bae, Soo Wan Kim, Chang Seong Kim, Se Hun Kim, Young Joon Ki, Dong Joon Kim, Suhyung Park

**Affiliations:** 1 Department of Intelligent Electronics and Computer Engineering, Chonnam National University, Gwangju 61186, Republic of Korea; jjh012601@gmail.com (J.J.); yhs9910@gmail.com (H.R.); lmomj1288@gmail.com (M.L.); 2 Department of Internal Medicine, Chonnam National University Hospital, Gwangju 61469, Republic of Korea; baedak@hanmail.net (E.H.B.); kdksw@hanmail.net (S.W.K.); laminion@hanmail.net (C.S.K.); 3 Department of Internal Medicine, Medical School, Chonnam National University, Gwangju 61469, Republic of Korea; 4 Department of Anesthesiology and Pain Medicine, Chosun University Hospital, Gwangju 61453, Republic of Korea; sepal1116@chosun.ac.kr (S.H.K.); darkstar97@chosun.ac.kr (Y.J.K.); 5 Department of Anesthesiology and Pain Medicine, Medical School, Chosun University, Gwangju 61452, Republic of Korea

**Keywords:** ultrasound needle segmentation, semi-supervised learning, unrolling optimization, dynamics-based deep learning, biopsy needle tracking

## Abstract

**Background/Objectives:** Ultrasound-guided biopsy is essential for accurate needle placement in minimally invasive procedures. However, the limited availability of labeled medical data significantly constrains the development of needle segmentation models, and existing semi-supervised approaches rely solely on data-driven consistency constraints without incorporating domain-specific dynamic properties of the imaging process. We propose a semi-supervised unrolling optimization framework that combines dynamics-based data consistency with self-training. **Methods:** (1) A null-space projection-based Analytic Model (A) layer is unrolled into the network to suppress background interference and enforce dynamics-based constraints derived from temporal ultrasound sequences, in which the background subspace is estimated via singular value decomposition (SVD) of consecutive frames and the needle signal is recovered by projecting the observed image onto the orthogonal complement of this subspace. (2) A self-training strategy leverages unlabeled ultrasound data by generating soft pseudo-labels from a pretrained teacher network to iteratively supervise the student model, which shares the same unrolling architecture and is jointly trained with a supervised loss on labeled data and a pseudo loss on unlabeled data. **Results:** Evaluation on an ultrasound needle segmentation dataset demonstrated that the proposed method achieved a Dice score of 0.7429±0.0489 and an IoU of 0.5967±0.0917, outperforming the supervised UnrollNet baseline (Dice: 0.7298±0.0518), Co-Training (U-Net) (Dice: 0.6104±0.0949), and Meta Pseudo Labels (Dice: 0.6426±0.1140). The proposed method also yielded a mean trajectory angle error of $0.66^{\circ }\pm 0.63^{\circ }$ and a tip position error of 0.95mm±0.74mm. **Conclusions:** These results indicate the potential of the proposed framework for ultrasound-guided needle tracking.

## 1. Introduction

Ultrasound (US)-guided percutaneous biopsy has been widely adopted as an essential procedure in minimally invasive surgery, enabling real-time imaging guidance for precise needle placement in hepatic, renal, thyroid, prostate and breast interventions [1,2]. Accurate and reliable identification of the biopsy needle within dynamic US image sequences is indispensable for reducing procedural complications, including inadvertent puncture of adjacent vasculature and critical anatomical structures, as well as for ensuring accurate tissue sampling. Despite its clinical utility, automated needle identification in US imaging remains a particularly challenging task owing to the inherent speckle noise of US acquisitions, acoustic shadowing artifacts, beam steering-induced intensity variations, and the high degree of visual similarity between the hyperechoic needle shaft and the surrounding soft tissue structures [3].

Conventional approaches to needle detection in US images have predominantly relied on background subtraction and geometric feature extraction techniques, in which temporal frame differencing, Hough transform-based line fitting, or oblique projection operators [4,5] are employed to isolate the needle signal from the cluttered tissue background. Although these methods offer computational efficiency and physical interpretability, their performance typically degrades under conditions of tissue motion, varying needle insertion angles, and non-stationary acoustic clutter, thereby potentially limiting their reliability in challenging clinical scenarios [6,7,8]. To address these limitations, deep learning (DL)-based methods have been developed that leverage encoder–decoder architectures such as U-Net [9] and its attention-gated variants to learn discriminative representations directly from US image data [10,11,12]. More recently, transformer-based architectures, including Swin U-Net [13] and TransUNet [14], have further extended DL-based segmentation by capturing long-range contextual dependencies, demonstrating improved boundary delineation in medical imaging tasks. Despite their promising segmentation performance, these fully supervised DL approaches are critically dependent on large-scale pixel-level annotations, which require substantial clinical expertise and are prohibitively costly to obtain, particularly for dynamic US sequences in which labeling demands scale with temporal resolution and inter-frame variability introduces clinician-dependent inconsistencies.

To alleviate the annotation burden, semi-supervised learning (SSL) methods jointly exploit a limited set of labeled samples and a substantially larger pool of unlabeled data [15], with Meta Pseudo Labels (MPL) [16] and co-training [17] as representative approaches. Nevertheless, existing SSL frameworks rely exclusively on data-driven consistency constraints and do not incorporate domain-specific dynamic properties of the imaging process. In US-guided needle tracking, the temporal evolution of needle and tissue signals is governed by well-characterized spatiotemporal dynamics, and disregarding this structure may yield pseudo-labels of insufficient fidelity under strong background clutter and pronounced tissue motion.

In this work, we develop a novel dynamics-based unrolling framework by embedding the dynamics-based signal model [18] of US needle imaging directly into the semi-supervised learning framework. To this end, the needle signal is represented by projecting the US sequence onto the orthogonal complement of the background subspace—a dynamics-guided constraint that inherently suppresses tissue interference, providing an additional supervisory signal grounded in the imaging dynamics rather than data statistics alone. A deep learning prior is incorporated as a learned regularizer within an unrolled optimization network [19,20,21], in which each iteration alternates between a U-Net-based regularization layer (R) and a dynamics-based Analytic Model layer (A). This enables each iterative refinement step to remain physically consistent throughout training and inference. To exploit unannotated US data, a self-training strategy with a pretrained teacher network is adopted. The framework is evaluated on an ultrasound needle dataset comprising 181 labeled and 2653 unlabeled images, with performance benchmarked against supervised baselines as well as competing SSL approaches, including MPL and co-training, across segmentation accuracy and clinical needle tracking metrics. The main contributions of this work are as follows:

- We introduce, to the best of our knowledge, the first dynamics-based unrolled deep network for biopsy needle identification in US imaging, in which domain-specific null-space projection constraints are embedded as an Analytic Model layer within the unrolling framework, enabling each optimization iteration to enforce background suppression grounded in the spatiotemporal signal model and thereby providing a structured inductive bias absent from purely data-driven architectures.
- We present the first semi-supervised approach to biopsy needle identification by employing a self-training strategy, wherein a pretrained teacher network generates soft pseudo-labels for unlabeled images that iteratively supervise the student model, thereby exploiting large volumes of unannotated US data without requiring additional clinical annotation effort.
- The proposed semi-supervised unrolled network outperforms both supervised baselines and competing SSL approaches including MPL and co-training in terms of region overlap (Dice score, IoU) and trajectory angle error, demonstrating the benefit of integrating dynamics-based inductive bias with semi-supervised learning for needle tracking.

## 2. Related Work

### 2.1. Semi-Supervised Learning for Medical Image Segmentation

Semi-supervised learning (SSL) aims to improve performance over supervised models by additionally exploiting structural information embedded in unlabeled data, while simultaneously leveraging labeled data to provide discriminative supervision [15,22,23]. A common SSL formulation introduces a regularization term into the supervised objective function to extract latent structure from unlabeled samples. From this perspective, SSL methods can be broadly categorized into pseudo labeling and consistency regularization. Pseudo labeling attempts to synthesize annotations for unlabeled samples [24], upon which the model is retrained under the supervised learning framework. Consistency regularization, in contrast, enforces invariance of the model’s output distribution under diverse input perturbations, without requiring explicit label generation.

### 2.2. Pseudo Labeling

Under vanilla pseudo labeling, the teacher is pretrained and held fixed throughout student training, where a teacher network generates pseudo-labels for unlabeled data to supervise a student network [25,26]. Despite its simplicity, fixing the teacher introduces a confirmation bias problem: if the teacher’s pseudo-labels are inaccurate, errors propagate to the student without correction, and the student may not surpass the teacher. To address this limitation, Meta Pseudo Labels (MPL) [16] casts pseudo-label generation as a bi-level optimization problem. Since the optimal student parameters depend implicitly on the teacher parameters via the pseudo-labels, the teacher can be optimized by minimizing the student’s loss on labeled data $L_{l}$. More specifically, student and teacher updates are executed sequentially at each training step:

(1) $$\begin{array}{cc} \theta_{S}^{\prime } & =\theta_{S}-\eta_{S}\nabla_{\theta_{S}}L_{u}\left(\theta_{T},\theta_{S}\right), \end{array}$$

(2) $$\begin{array}{cc} \theta_{T}^{\prime } & =\theta_{T}-\eta_{T}\nabla_{\theta_{T}}L_{l}\;\left(\theta_{S}-\eta_{S}\nabla_{\theta_{S}}L_{u}\left(\theta_{T},\theta_{S}\right)\right), \end{array}$$

where $\eta_{S}$ and $\eta_{T}$ are the learning rates for the student and teacher, respectively, and the student’s gradient $\nabla_{\theta_{S}}L_{u}$ is reused in the teacher’s update step. The key distinction from vanilla pseudo labeling is that the teacher parameters are continuously adapted via the student’s performance feedback on labeled data, enabling iterative refinement rather than relying on a fixed, potentially biased teacher.

### 2.3. Consistency Regularization

The central premise of consistency regularization is that a well-generalizing model should produce stable, invariant predictions for a given unlabeled sample regardless of the specific stochastic perturbation applied. The unsupervised regularization term is formulated as a consistency loss between model predictions on perturbed versions of the same unlabeled sample:

(3) $$L=L_{\sup}+\lambda L_{\mathrm{consistency}},$$

where $L_{\sup}$ is the supervised segmentation loss on labeled pairs, and λ>0 is a weighting coefficient that balances the two objectives. Among consistency-based methods, the Mean Teacher model [27] enforces consistency between a student network and a teacher whose parameters are updated as an exponential moving average (EMA) of the student’s weights:

(4) $$\theta_{T}\leftarrow \alpha \theta_{T}+\left(1-\alpha \right)\;\theta_{S},$$

where α∈[0,1) is the EMA decay coefficient. By decoupling the teacher from direct gradient updates, Mean Teacher produces smoother, more stable prediction targets for the consistency loss, thereby reducing the risk of error accumulation from noisy self-predictions. Similar consistency-based strategies have also been applied to ultrasound image segmentation [28,29].

Despite their empirical success, consistency regularization methods are inherently data-centric and provide no mechanism to incorporate domain-specific dynamic constraints, which motivates the dynamics-based formulation adopted in this work.

## 3. Materials and Methods

**Figure 1:**
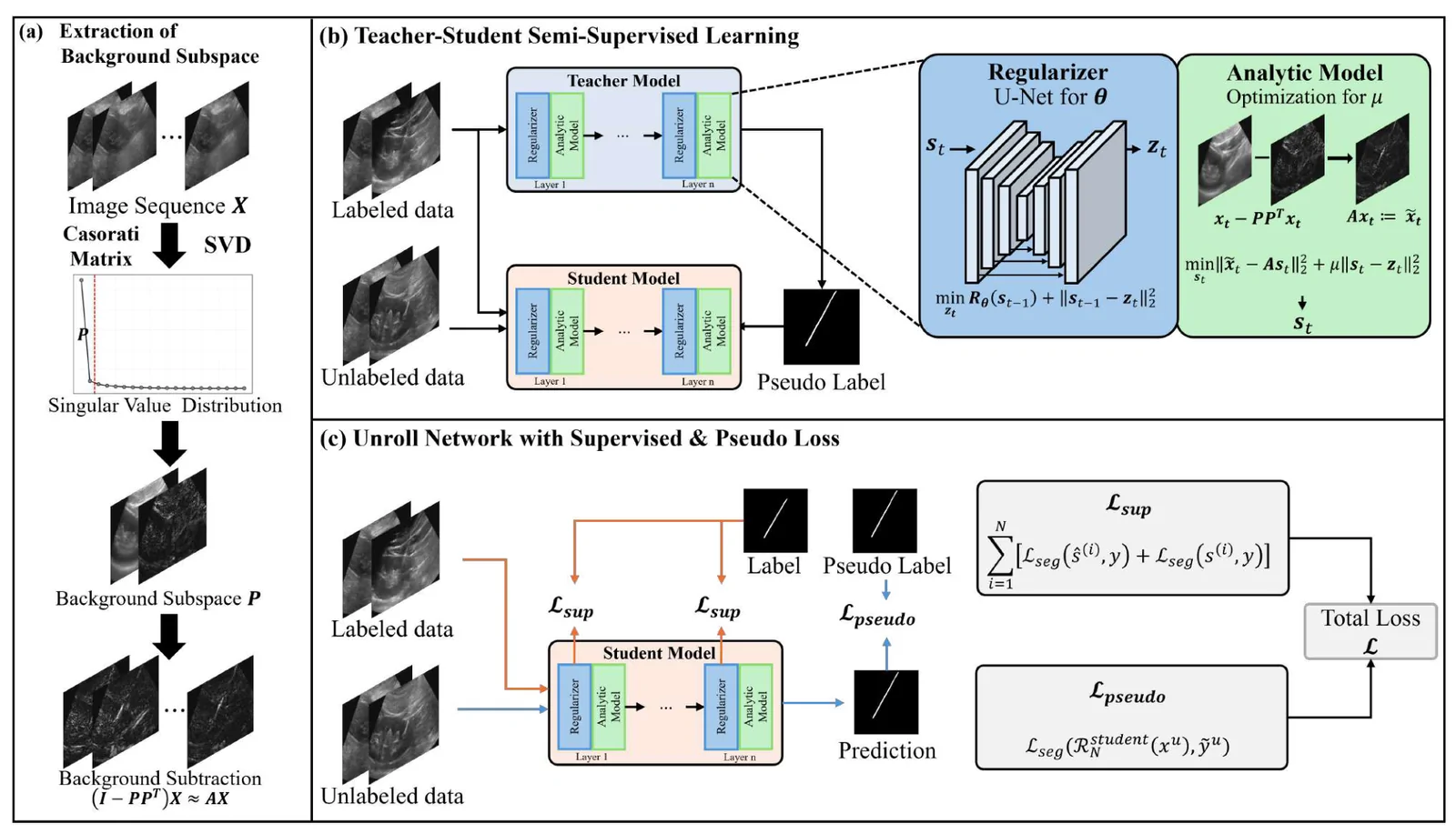
The graphic illustration of the proposed semi-supervised unrolled network for biopsy needle identification in ultrasound. (**a**) Background subspace *P* is extracted from the input image sequence via SVD of the Casorati matrix, and used to compute the background-subtracted image ${\tilde{x}}_{t}=\left(I-PP^{T}\right)x_{t}$. (**b**) The self-training pipeline consists of a fixed teacher UnrollNet that generates soft pseudo-labels ${\tilde{y}}^{u}$ for unlabeled images $x^{u}$, and a student UnrollNet jointly supervised by the ground-truth segmentation loss $L_{\sup}$ on labeled data and the pseudo-label loss $L_{\mathrm{pseudo}}$ on unlabeled data. Each unrolling iteration alternates between a U-Net-based regularization layer (R) and a dynamics-based Analytic Model layer (A). (**c**) Note that the teacher network parameters are held fixed throughout student training, and pseudo-label supervision is applied exclusively to the final-layer output $R_{N}^{\mathrm{student}}\left(x^{u}\right)$ of the student UnrollNet, while labeled-data supervision propagates through all intermediate R and A outputs.

An overview of the proposed semi-supervised dynamics-based unrolling framework is illustrated in Figure 1, comprising background subspace extraction via SVD, the teacher-student self-training pipeline, and the unrolled R+A network trained with supervised and pseudo losses.

### 3.1. Problem Formulation

Based on the spatiotemporal signal model $x_{t}$ proposed by Park et al. [18], the dynamic ultrasound image at each time frame can be written as a superposition of the background tissue signal and the foreground needle signal:

(5) $$x_{t}=l_{t}+s_{t}+n_{t}$$

where $x_{t}\in R^{n_{s}\times 1}$ is the observed image, $l_{t}\in R^{n_{s}\times 1}$ is the background tissue component, $s_{t}\in R^{n_{s}\times 1}$ is the sparse needle signal, and $n_{t}$ denotes unstructured noise. The background component $l_{t}$ can be represented using a low-dimensional subspace estimated from consecutive frames via singular value decomposition (SVD):

(6) $$l_{t}=U_{t,\|}\alpha_{t}$$

where $U_{t,\|}\in R^{n_{s}\times r}$ spans the column space of the background matrix constructed from the first $T^{\prime }$ consecutive frames of the ultrasound sequence, $L=[l_{1},l_{2},\ldots ,l_{T^{\prime }}]$, and $\alpha_{t}$ is the corresponding coordinate coefficient vector. Since the needle signal $s_{t}$ is incoherent with the background subspace, it lies in the null space of $U_{t,\|}$ and can be recovered via null-space projection:

(7) $$U_{t,⊥}U_{t,⊥}^{H}x_{t}\approx s_{t}$$

where $U_{t,⊥}$ spans the null space of $U_{t,\|}$. In practice, we implement the null-space projection as $\left(I-PP^{T}\right)$, where *P* is the matrix of background basis vectors obtained from SVD, and $PP^{T}$ projects onto the background subspace. The projected image is denoted as:

(8) $${\tilde{x}}_{t}=\left(I-PP^{T}\right)x_{t}$$

Given this formulation, needle segmentation is cast as a regularized inverse problem:

(9) $${\hat{s}}_{t}=\arg\min_{s_{t}}\frac{1}{2}{∥{\tilde{x}}_{t}-A_{t}s_{t}∥}_{2}^{2}+\lambda R\left(s_{t}\right)$$

where $A_{t}=I-PP^{T}$ is the null-space projection operator and R(·) is a regularization term that promotes sparsity of the needle signal.

To decouple the data fidelity and regularization terms, we introduce an auxiliary variable $z_{t}$ and reformulate via variable splitting:

(10) $$\begin{array}{cc} \; & \min_{s_{t}}\frac{1}{2}∥{\tilde{x}}_{t}-A_{t}s_{t}{∥}_{2}^{2}+\frac{\mu }{2}{∥s_{t}-z_{t}∥}_{2}^{2} \end{array}$$

(11) $$\begin{array}{cc} \; & \min_{z_{t}}\lambda R\left(z_{t}\right)+\frac{\mu }{2}{∥s_{t}-z_{t}∥}_{2}^{2} \end{array}$$

The $s_{t}$-subproblem has a closed-form solution. Setting the gradient to zero:

(12) $$\begin{array}{cc} \; & -A_{t}^{T}\left({\tilde{x}}_{t}-A_{t}s_{t}\right)+\mu \left(s_{t}-z_{t}\right)=0 \end{array}$$

(13) $$\begin{array}{cc} \; & \left(A_{t}^{T}A_{t}+\mu I\right)s_{t}=A_{t}^{T}{\tilde{x}}_{t}+\mu z_{t} \end{array}$$

Since $A_{t}=I-PP^{T}$ is symmetric and idempotent ($A_{t}^{T}=A_{t}$, $A_{t}^{2}=A_{t}$), we have $A_{t}^{T}A_{t}=A_{t}$, simplifying to:

(14) $$s_{t}={\left(A_{t}+\mu I\right)}^{-1}\left(A_{t}{\tilde{x}}_{t}+\mu z_{t}\right)$$

This linear system is solved efficiently using the conjugate gradient (CG) method. The $z_{t}$-subproblem has no closed-form solution for general R(·), and is instead handled by a learned network as described in the next section.

### 3.2. Dynamics-Based Unrolling Network

To solve the above inverse problem in a data-driven manner, we adopt an unrolling optimization framework [21,30,31] that maps the iterative solution steps to a sequence of learnable network layers. Each unrolling iteration consists of two alternating steps: a regularization network (R) and an Analytic Model (A).

#### 3.2.1. Regularization Network (R)

At each iteration *i*, a U-Net-based regularization network $R_{i}$ takes the current estimate as input and produces an updated estimate:

(15) $${\hat{s}}^{\left(i\right)}=R_{i}\left(s^{\left(i-1\right)}\right)$$

Each iteration uses an independent set of network parameters, allowing the model to learn iteration-specific features.

#### 3.2.2. Analytic Model (A)

Following the regularization step, a dynamics-based data consistency layer—referred to as the Analytic Model (A)—enforces the null-space projection constraint by solving:

(16) $$s^{\left(i\right)}=\arg\min_{s}\frac{1}{2}∥\tilde{x}{-As∥}_{2}^{2}+\frac{\mu^{\left(i\right)}}{2}{∥s-{\hat{s}}^{\left(i\right)}∥}_{2}^{2}$$

where $\mu^{\left(i\right)}$ is a learnable parameter that balances the data fidelity and regularization terms at each iteration. This quadratic problem is solved efficiently using the CG method. The overall unrolling process alternates between R and A for *N* iterations, producing intermediate outputs ${\left\{\left({\hat{s}}^{\left(i\right)},s^{\left(i\right)}\right)\right\}}_{i=1}^{N}$ at each step.

### 3.3. Semi-Supervised Self-Training

The availability of labeled ultrasound data is limited in practice, as pixel-level annotation requires clinical expertise. To leverage unlabeled data, we adopt a self-training framework in which a pretrained teacher network generates pseudo-labels for unlabeled images to supervise a student network.

#### 3.3.1. Teacher Network

The teacher network is initialized from a supervised UnrollNet pretrained on the labeled dataset and its parameters are fixed throughout training. Given an unlabeled image $x^{u}$, the teacher generates a soft pseudo-label:

(17) $${\tilde{y}}^{u}=R_{N}^{\mathrm{teacher}}\left(x^{u}\right)$$

where $R_{N}^{\mathrm{teacher}}$ denotes the final R output of the teacher network. Soft pseudo-labels are used instead of hard binary labels to retain the prediction confidence of the teacher.

#### 3.3.2. Student Network

The student network shares the same UnrollNet architecture as the teacher and is trained on both labeled and unlabeled data. The training objective consists of two loss terms:

(18) $$L=L_{\sup}+\lambda_{p}L_{\mathrm{pseudo}}$$

The supervised loss $L_{\sup}$ is computed on labeled data using the ground truth mask *y*, applied to all intermediate R and A outputs, where ${\hat{s}}^{\left(i\right)}$ and $s^{\left(i\right)}$ denote the R and A outputs at iteration *i*, respectively:

(19) $$L_{\sup}=\sum_{i=1}^{N}\left[L_{\mathrm{seg}}\;\left({\hat{s}}^{\left(i\right)},y\right)+L_{\mathrm{seg}}\;\left(s^{\left(i\right)},y\right)\right]$$

The pseudo loss $L_{\mathrm{pseudo}}$ supervises the student using the teacher’s soft pseudo-labels on unlabeled data:

(20) $$L_{\mathrm{pseudo}}=L_{\mathrm{seg}}\;\left(R_{N}^{\mathrm{student}}\;\left(x^{u}\right),\;{\tilde{y}}^{u}\right)$$

where $\lambda_{p}=0.7$ is the pseudo loss weight. As the segmentation loss $L_{\mathrm{seg}}$, we use mean squared error (MSE) loss.

### 3.4. Dataset

We evaluate our method on an ultrasound needle segmentation dataset collected from renal biopsy procedures at Chonnam National University Hospital and Chosun University Hospital, each contributing approximately 50% of the dataset, approved by the Institutional Review Board (CNUH-2021-446, Chosun 2024-10-001). Images were acquired using two ultrasound systems from the same vendor, a Philips iU22 and a Philips CX50 (Philips Ultrasound, Bothell, WA, USA), each equipped with a curved array transducer, at a resolution of 640×480 pixels and a maximum frame rate of 30 frames per second. All biopsy procedures were performed by board-certified physicians using a spring-loaded 18-gauge biopsy needle inserted at depths of approximately 10–15 cm from the skin surface at insertion angles between $30^{\circ }$ and $60^{\circ }$. The dataset consists of 181 labeled images and 2653 unlabeled images acquired during ultrasound-guided percutaneous renal biopsy. The labeled images are split into a training set of 148 images and a validation set of 18 images, with 15 images reserved for testing. To further assess the reliability of the evaluation, an additional test set of 176 labeled images was annotated from videos different from those of the original labeled set, and was used only for evaluation (Section 4.3).

Unlike common SSL benchmarks that artificially withhold labels from a fully annotated dataset, the unlabeled images in our dataset were unannotated at the time of acquisition, reflecting a realistic clinical scenario in which pixel-level annotation is available only for a small subset of collected data. The unlabeled images are used solely for pseudo-label supervision and contain no annotations. The split was performed at the video level, such that no video contributes frames to more than one subset. Patient identifiers were not recorded. Labeling was performed by experienced nephrologists and anesthesiologists using the CVAT platform [32]. Disagreements were then resolved through mutual checking and review among them.

### 3.5. Implementation Details

The proposed framework was implemented in PyTorch 2.6.0 with CUDA 12.4 on an NVIDIA RTX 3090 GPU (24 GB VRAM) running Ubuntu 18.04.6 LTS. Each unrolling iteration comprises an independent U-Net serving as the regularization network and a CG-based A layer solved over 10 conjugate gradient iterations, and the number of unrolling iterations was set to N=5 for both the teacher and the student (see Section 4.1.1). The background subspace matrix *P* was precomputed by applying SVD to a Casorati matrix constructed from the first $T^{\prime }=20$ consecutive ultrasound frames per sequence, retaining the two dominant left singular vectors as the background basis (r=2). The matrix *P* was kept fixed during training and shared across all frames of the sequence. All input images were resized to 256×256 pixels prior to training. The teacher was a supervised UnrollNet (N=5) that remained frozen during self-training, and the student was randomly initialized (i.e., not initialized from the teacher weights). The model was trained for up to 100 epochs using the Adam optimizer with a learning rate of $1\times 10^{-4}$ and a batch size of 4. Each iteration used one labeled and one unlabeled mini-batch, with an epoch defined as one pass over the labeled set. The learning rate was halved by ReduceLROnPlateau (factor 0.5, patience 10) on the validation Dice. Early stopping with a patience of 30 epochs was applied on the validation Dice of the final A-layer output, and the checkpoint with the best validation Dice was used for testing. Data augmentation was applied to the labeled training set via pre-computed rotations of $\pm 25^{\circ }$ and $\pm 40^{\circ }$, yielding 5× the original number of labeled samples. The source code is available at https://github.com/jjh012601-imispl/UnrollNet-needle (accessed on 16 October 2026).

### 3.6. Evaluation Metrics

Segmentation performance is evaluated using the Dice similarity coefficient, Intersection over Union (IoU), and 95th percentile Hausdorff distance (HD95), which collectively assess overlap accuracy and boundary precision. For clinical relevance, needle tracking metrics are additionally reported, including trajectory angle error and tip-position error. For trajectory estimation, the needle axis was fitted to the predicted segmentation mask using the Hough transform (Canny edges, threshold 30, minimum line length 50, maximum gap 20), with a RANSAC line fit (residual threshold 2, 1000 trials) as a fallback. The tip was defined as the deepest foreground pixel along the fitted axis. The trajectory angle error is the absolute difference between the angles of the predicted and ground-truth axes, and the tip-position error is the Euclidean distance between the predicted and ground-truth tips, converted to millimeters using a pixel spacing of 0.1mm/pixel.

## 4. Results

### 4.1. Ablation Study

#### 4.1.1. Effect of the Number of Unrolling Iterations

**Figure 2:**
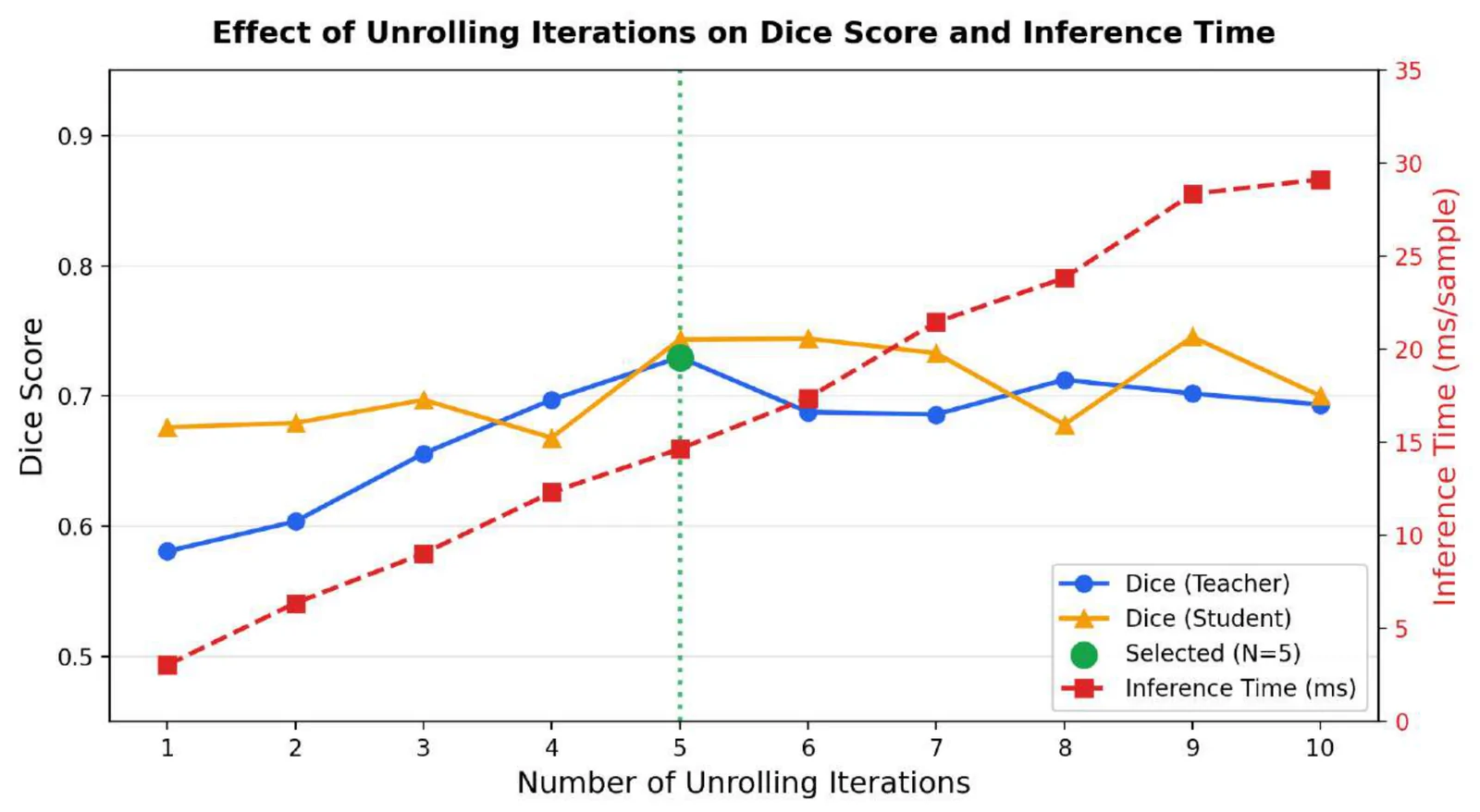
Effect of the number of unrolling layers *N* on Dice score and inference time. Dice scores of the supervised teacher (solid blue line) and the SSL student (solid orange line) are plotted on the left axis, and network forward-pass inference time in ms/sample (dashed red line) on the right axis, as a function of N∈{1,2,…,10}. The student was trained with the teacher fixed at N=5. Since the teacher and the student share the same architecture, the inference time is common to both. Note that the teacher achieves its peak Dice of 0.73 at N=5, after which performance exhibits non-monotonic behavior while inference time increases monotonically, and the student also shows comparable performance at N=5, choosing the number of layers N=5 as the default configuration for both networks.

The number of unrolling iterations *N* was determined by evaluating the supervised teacher model across N∈{1,2,…,10}, with performance measured by Dice score and inference time on the test set. Inference time refers to the network forward pass only (*N* unrolled R–A iterations with 10 CG steps on a 256×256 input) and excludes the one-time SVD-based background subspace estimation, data loading, and trajectory/tip estimation. As shown in Figure 2, the Dice score of the teacher peaks at N=5 while inference time increases monotonically. For the student, we also trained the model with each N∈{1,2,…,10} while keeping the teacher fixed at N=5, and N=5 was again among the best-performing settings. Based on these observations, N=5 was selected as the default configuration for both the teacher and the student.

#### 4.1.2. Effect of Labeled Data Proportion on Segmentation Performance

**Figure 3:**
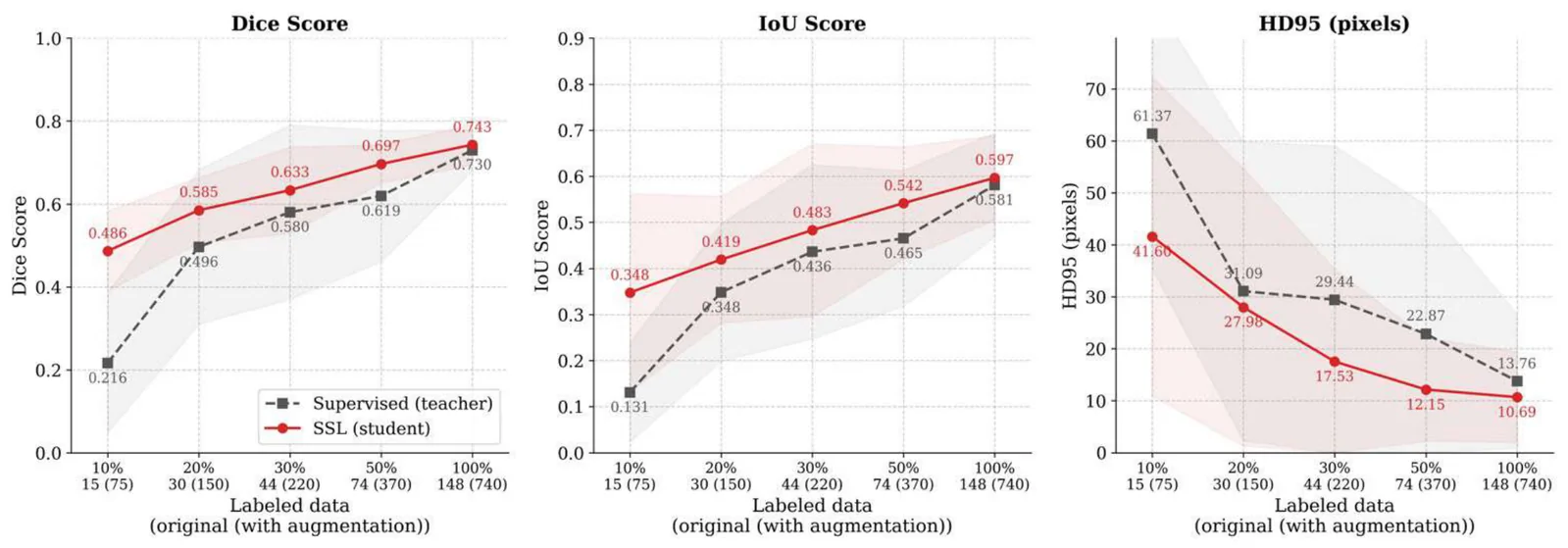
Segmentation performance of the SSL student and the supervised UnrollNet trained on the same labeled data, as a function of labeled data proportion. Dice, IoU, and HD95 are shown for 10%, 20%, 30%, 50%, and 100% of the training set (15, 30, 44, 74, and 148 original images; 75, 150, 220, 370, and 740 after augmentation), with shaded bands indicating ± one standard deviation. At 50% labeled data, the SSL student achieves a Dice of 0.6965 and an IoU of 0.5418, approaching the fully supervised UnrollNet baseline (Dice: 0.7298) trained with 100% labeled data, demonstrating the data efficiency of the self-training strategy.

To analyze the data efficiency of the proposed method, we train the model with varying proportions of labeled data (10%, 20%, 30%, 50%, 100%) while keeping the full unlabeled set fixed. Table 1 and Figure 3 summarize the segmentation performance under varying proportions of labeled data. Performance improves consistently with the proportion of labeled data across Dice, IoU and HD95, with Dice increasing from 0.4865 at 10% to 0.7429 at 100%. Notably, at 50% labeled data, the proposed method already achieves a Dice of 0.6965, approaching the supervised UnrollNet baseline (0.7298) trained without any unlabeled data. At every proportion, the SSL student achieved a higher Dice than the supervised UnrollNet trained with the same labeled data.

#### 4.1.3. Effect of the Analytic Model (A)

**Figure 4:**
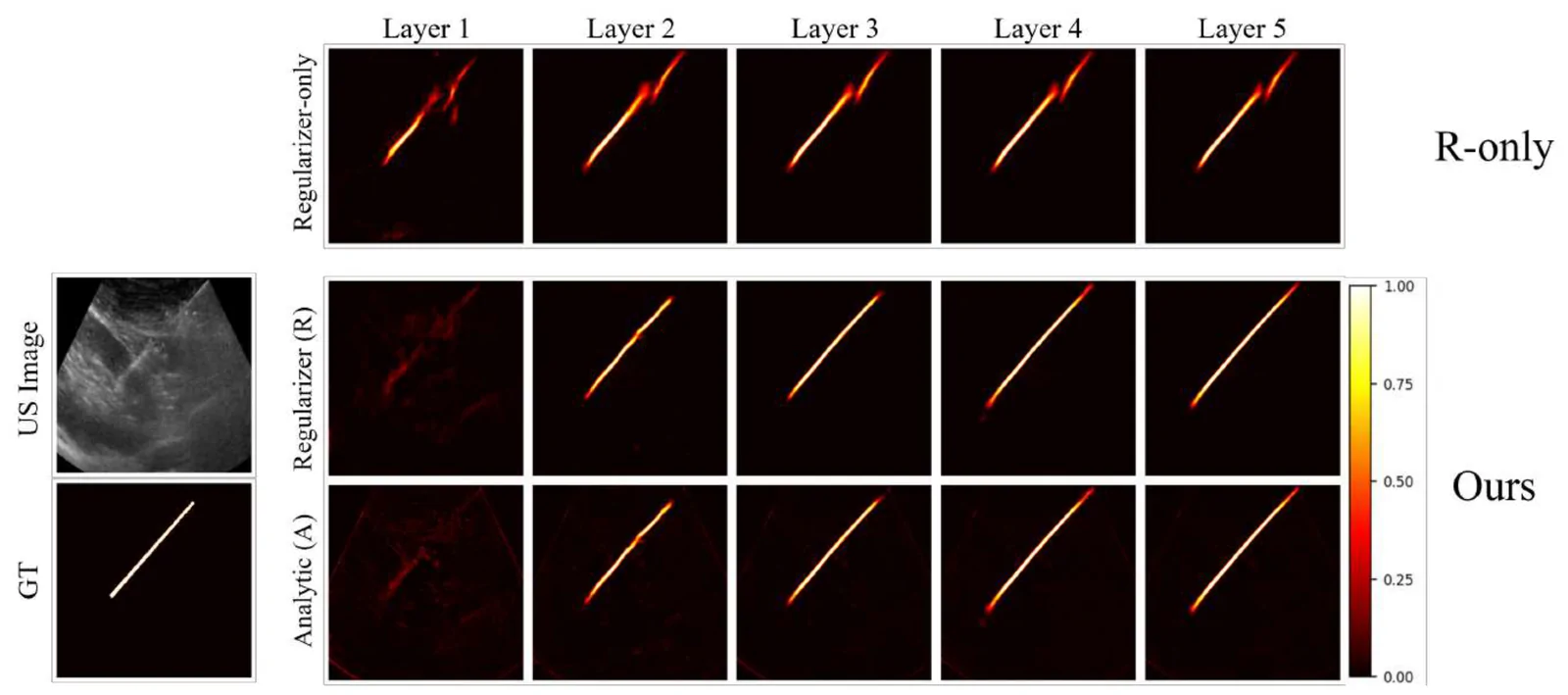
Visualization of intermediate outputs across N=5 unrolling layers for a case in which the biopsy needle is clearly visible under favorable imaging conditions. Columns correspond to Layers 1–5 of the unrolling sequence. All outputs are visualized as soft probability maps in the range [0, 1]. Top row: the R-only model (without the Analytic Model); middle row: the Regularizer (R) outputs of the proposed R+A model; bottom row: the Analytic Model (A) outputs of the proposed R+A model. The ground-truth (GT) mask is shown at the left for reference. Note that the R-only model generates a discontinuous, spatially fragmented needle response despite favorable imaging conditions, while the R+A model enforces spatially consistent activations through dynamics-based null-space projection, yielding a coherent and continuous needle delineation across all unrolling layers.

**Figure 5:**
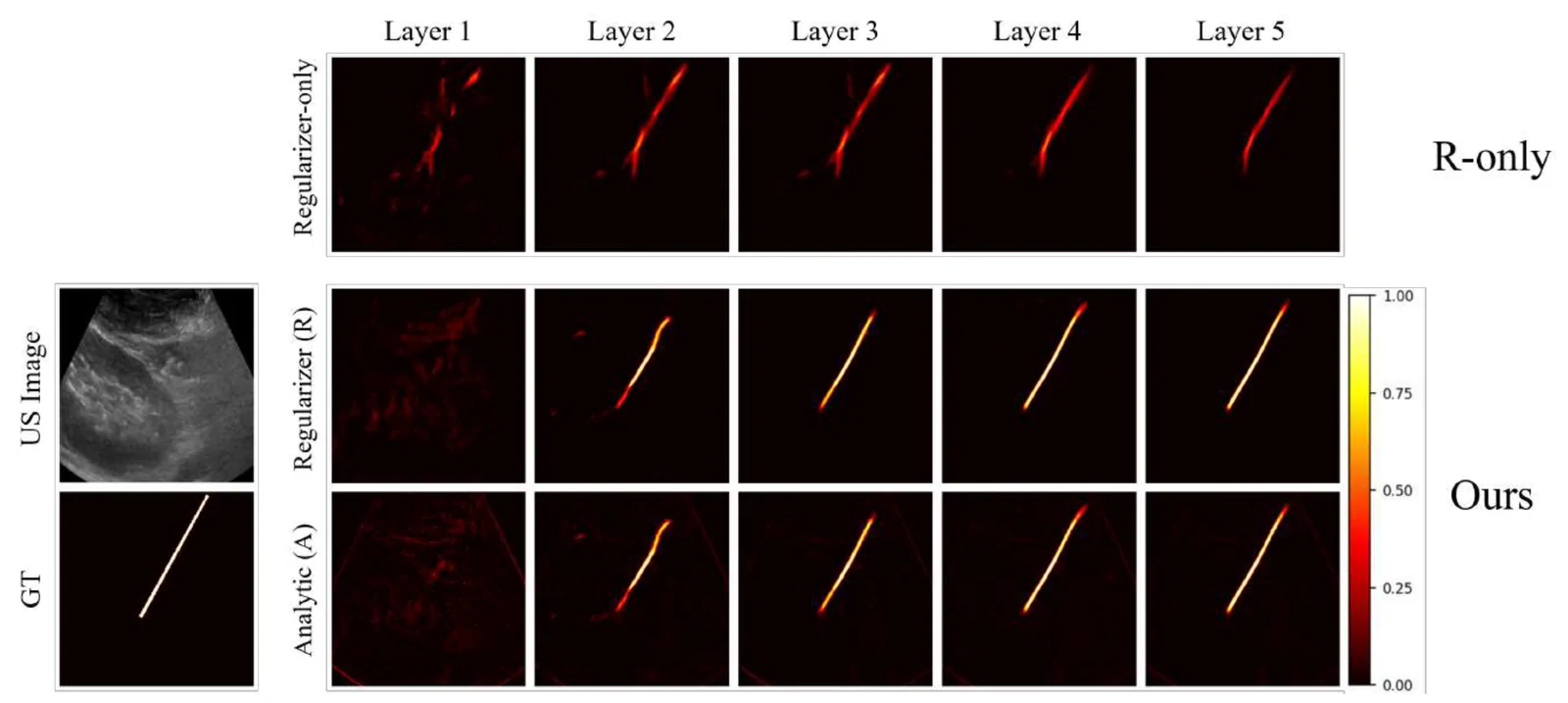
Visualization of intermediate outputs across N=5 unrolling layers for a challenging case in which the biopsy needle is poorly visible. Columns correspond to Layers 1–5 of the unrolling sequence. All outputs are visualized as soft probability maps in the range [0, 1]. Top row: the R-only model (without the Analytic Model); middle row: the Regularizer (R) outputs of the proposed R+A model; bottom row: the Analytic Model (A) outputs of the proposed R+A model. The ground-truth (GT) mask is shown at the left for reference. Note that the R-only model produces diffuse, spatially scattered activations across all five iterations, failing to suppress background tissue interference, while the R+A model rapidly localizes the needle signal following the first Analytic Model layer, with activations converging to a spatially coherent needle trajectory in subsequent layers.

**Figure 6:**
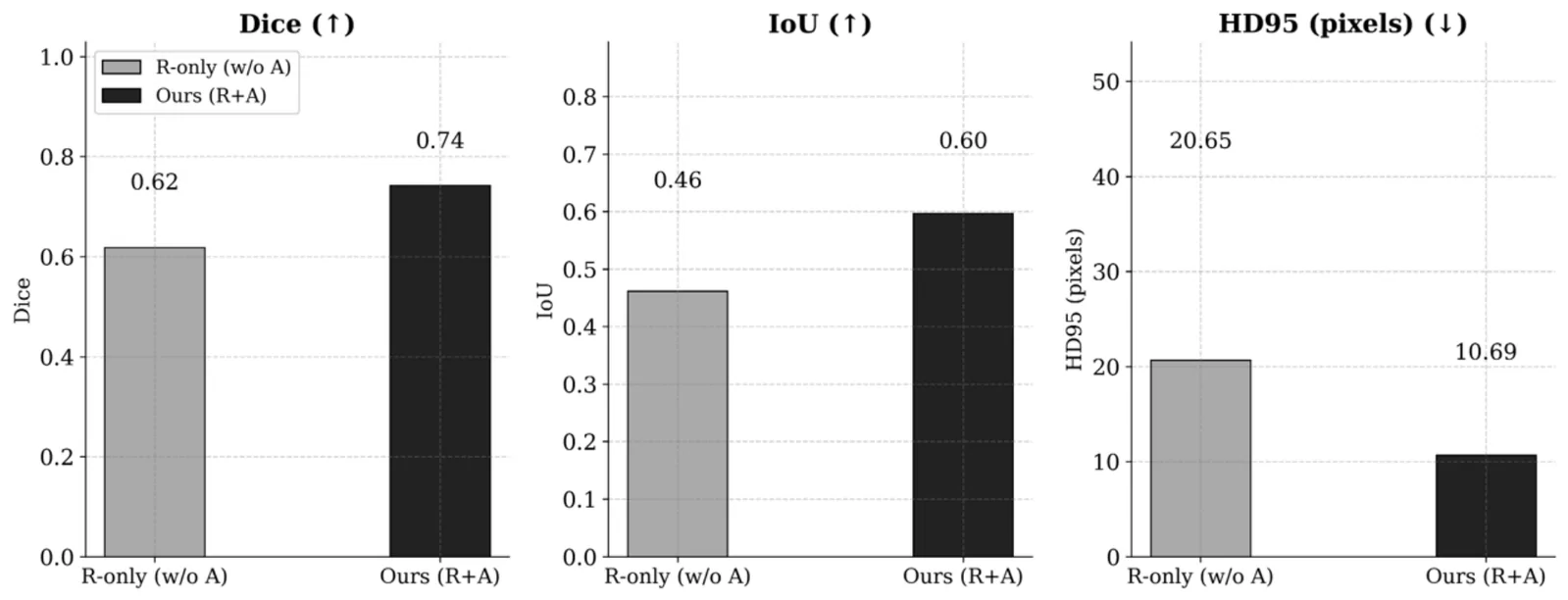
Quantitative comparison between the R-only model (without the Analytic Model) and the proposed R+A model across three segmentation metrics: Dice, IoU, and HD95 are shown as bar plots for both configurations. Note that removing the Analytic Model (A) layer results in consistent degradation across all metrics, with Dice decreasing from 0.7429 to 0.6184 and HD95 increasing from 10.69 to 20.65 pixels, confirming that the dynamics-based null-space projection enforced by the A layer provides an essential inductive bias for both segmentation accuracy and boundary delineation precision. ↑ indicates that higher values are better, and ↓ indicates that lower values are better.

To validate the contribution of the Analytic Model (A), we train a variant in which the A layer is removed and the R output is directly fed into the next R layer (R-only). Table 2 summarizes the results. Removing the A layer results in a Dice decrease from 0.7429 to 0.6184 and an HD95 increase from 10.69 to 20.65 pixels. As illustrated in Figure 4, Figure 5 and Figure 6, the R-only model produces discontinuous or diffuse activations across all unrolling layers, whereas the R+A model rapidly converges to a coherent needle trajectory following the first A layer.

### 4.2. Comparison with State of the Art

To benchmark the performance of the proposed method, four competing methods are evaluated: two supervised baselines and two semi-supervised baselines. The supervised baselines include (a) U-Net [9], a standard encoder–decoder segmentation architecture trained exclusively on labeled data, and (b) UnrollNet (sup.), the proposed dynamics-guided unrolling network trained under full supervision without unlabeled data, which serves as the supervised baseline reference. The semi-supervised baselines include (c) Meta Pseudo Labels (MPL) [16], which introduces a bi-level teacher-student optimization to reduce confirmation bias in pseudo-label generation, and (d) Co-Training [17], which employs two networks (U-Net [9] and Swin U-Net [13]) trained in parallel with mutual pseudo-label supervision to encourage view-consistent predictions.

Table 3 summarizes the quantitative comparison of segmentation and needle tracking performance across all supervised and semi-supervised methods. The proposed method achieves the highest Dice (0.7429±0.0489) and IoU (0.5967±0.0917), outperforming all competing semi-supervised baselines in region overlap. Compared to UnrollNet (sup.), which serves as the supervised baseline reference, the proposed method yields an incremental improvement of +0.0131 in Dice (from 0.7298 to 0.7429), suggesting that the semi-supervised self-training strategy can exploit unlabeled data beyond the supervised model. Among the compared methods, MPL achieves the lowest HD95 (8.02 pixels) and tip-position error (0.65mm±0.61mm), at the cost of substantially reduced region overlap (Dice of 0.6426), while Co-Training (Swin) shows the lowest Dice (0.5495) and IoU (0.3892). It is noted that the proposed method achieves the lowest trajectory angle error ($0.66^{\circ }\pm 0.63^{\circ }$ vs. $1.55^{\circ }$ for MPL) together with markedly higher segmentation overlap (Dice: 0.7429 vs. 0.6426), indicating the benefit of combining dynamics-based null-space projection with semi-supervised self-training. An extended evaluation on 176 additional test images, which compares the supervised UnrollNet and the SSL student using per-sample distributions of all five metrics, is presented in Section 4.3 (Table 4).

**Figure 7:**
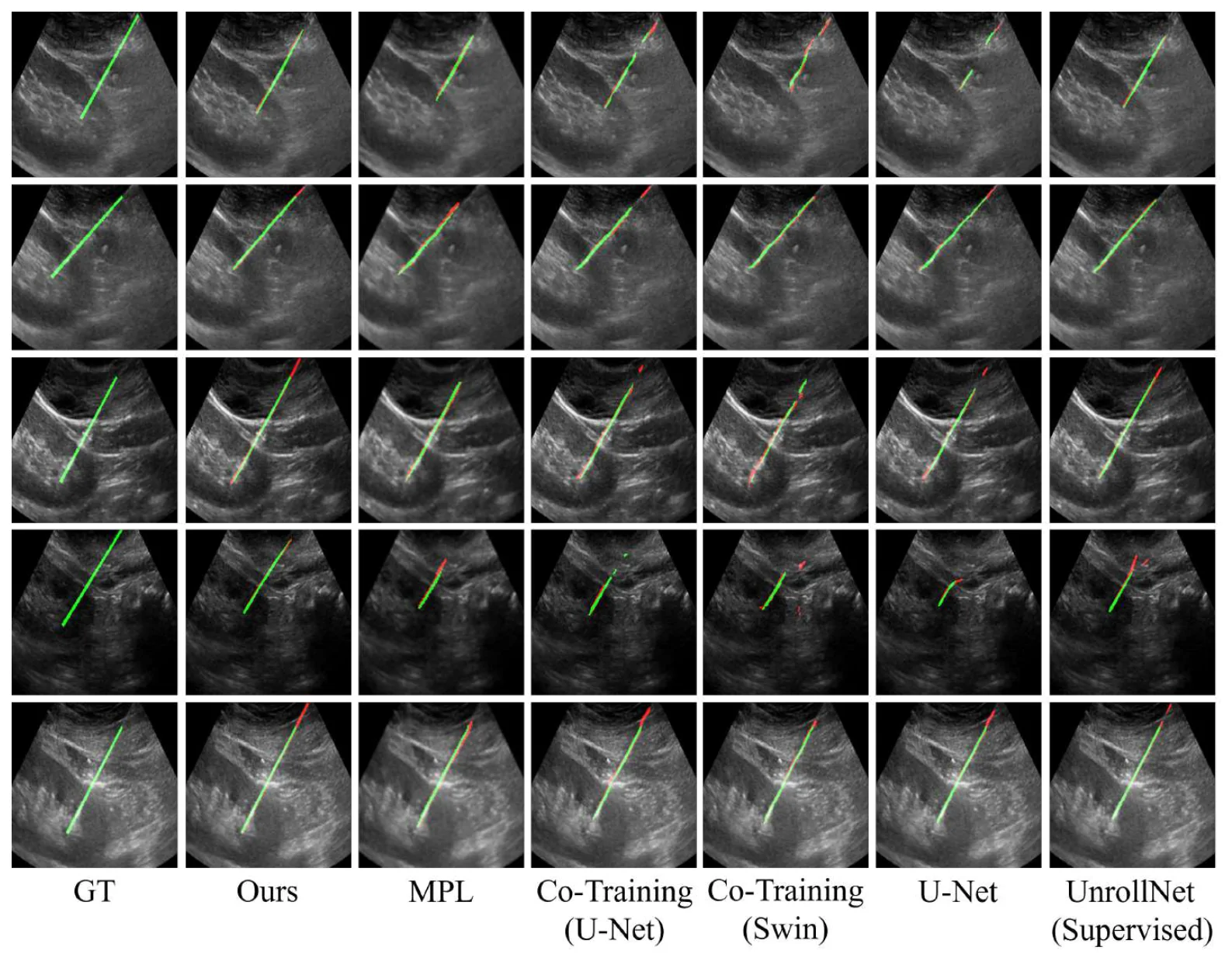
Qualitative comparison of biopsy needle segmentation results on representative test cases acquired during ultrasound-guided percutaneous renal biopsy. Each row corresponds to a distinct test sample; columns correspond to ground truth (GT), the proposed method (Ours), Meta Pseudo Labels (MPL), Co-Training (U-Net), Co-Training (Swin U-Net), supervised U-Net, and supervised UnrollNet, respectively. Predicted segmentation masks are overlaid on the original ultrasound images, with true positive regions shown in green and false positive regions in red. Note that MPL and Co-Training (Swin U-Net) exhibit frequent false positives and fragmented needle delineation, particularly in test cases with low needle-to-background contrast (rows 1 and 4), while our method produces generally the most continuous and accurate needle trajectories across all test cases.

The qualitative results show consistent improvement of the proposed method compared to all supervised and semi-supervised methods, as shown in Figure 7. Each column corresponds to ground truth (GT), proposed method, MPL, Co-Training (U-Net), Co-Training (Swin), U-Net, and UnrollNet (Supervised) with each row presenting an independent test image. Note that the proposed method consistently produces geometrically coherent, spatially continuous needle predictions across all five cases, while U-Net exhibits trajectory distortion and irregular boundary delineation particularly under low needle-to-background contrast conditions. On the other hand, both MPL and Co-Training variants display increased false-positive responses and shaft fragmentation in acoustically challenging regions, likely attributable to pseudo-label noise accumulated in the absence of dynamics-based background suppression. Additional qualitative results in video format, covering normal, low-visibility, and dark-contrast imaging conditions, are provided in the Supplementary Materials (Videos S1–S3).

### 4.3. Extended Evaluation on Additional Test Images

**Figure 8:**
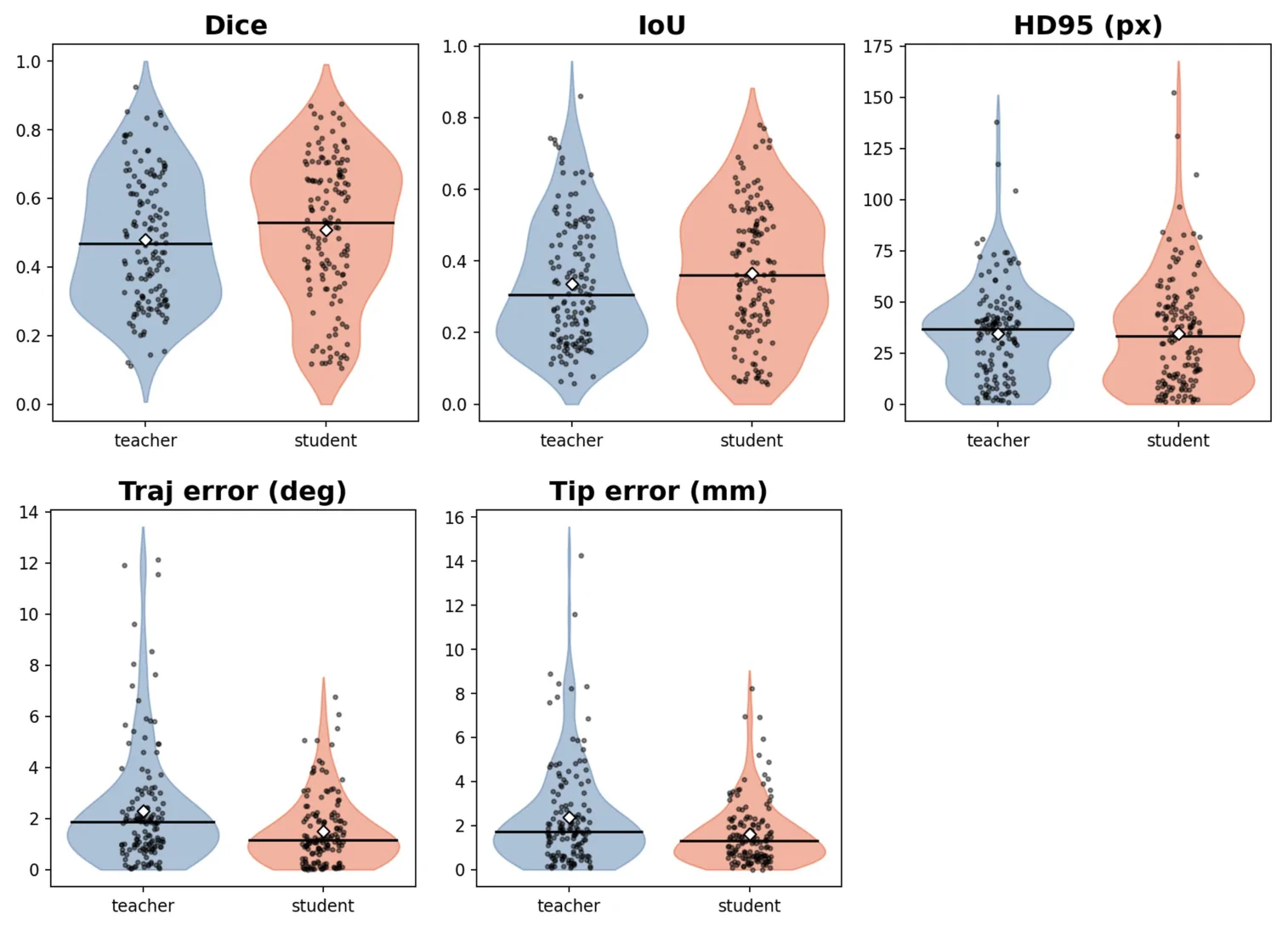
Violin plots of Dice, IoU, HD95, trajectory error, and tip-position error for the supervised UnrollNet (teacher, blue) and the SSL student (red) on the extended test set (N=176). Note that the SSL student shows higher median Dice (0.529 vs. 0.466) and IoU (0.360 vs. 0.304), and lower median HD95 (33.2 vs. 36.7 pixels), trajectory error ($1.14^{\circ }$ vs. $1.84^{\circ }$), and tip error (1.25mm vs. 1.70mm) than the supervised UnrollNet.

Since the original test set of 15 images limits the reliability of the reported results, the supervised UnrollNet and the proposed SSL student were additionally evaluated on the extended test set described in Section 3.4, which was annotated from videos different from those of the original labeled set. To demonstrate the robustness across individual samples rather than relying on mean values alone, the per-sample distributions of all five metrics are compared in Figure 8, and the mean ± standard deviation of each metric is summarized in Table 4. The SSL student showed a higher median Dice (0.529 vs. 0.466) and IoU (0.360 vs. 0.304), and a lower median HD95 (33.2 vs. 36.7 pixels), trajectory error ($1.14^{\circ }$ vs. $1.84^{\circ }$), and tip error (1.25mm vs. 1.70mm) than the supervised UnrollNet, whereas the mean HD95 was comparable between the two models.

## 5. Discussion

Our results suggest that the combination of dynamics-based null-space projection and semi-supervised self-training improves segmentation overlap and trajectory angle accuracy in ultrasound-guided biopsy needle tracking, highlighting the role of domain-specific inductive bias in compensating for annotation scarcity compared with purely data-driven semi-supervised approaches.

The performance improvement attributable to the A layer can be understood through its role in the unrolled optimization. The null-space projection operator $A_{t}=I-PP^{T}$ projects the observed ultrasound sequence onto the orthogonal complement of the background subspace estimated by SVD, thereby recovering the sparse needle signal $s_{t}$ from the clutter-dominated measurement $x_{t}$. By enforcing this dynamics-based constraint at each layer, the A layer continuously corrects the optimization trajectory toward physically consistent solutions, reducing the burden on the learned R layer and stabilizing convergence under the limited supervision. It is noted that this correction provides a richer inductive bias than data-driven consistency, as the spatiotemporal structure of the needle signal is governed by dynamics that are analytically characterized from the imaging model. As shown in Figure 4, Figure 5 and Figure 6, the R+A model rapidly localizes the needle signal after the first A layer and converges to a sharp, coherent activation pattern in subsequent iterations, whereas the R-only model produces diffuse or discontinuous predictions throughout all layers. This dynamics-based inductive bias comes at the cost of a dependency on the availability of consecutive temporal frames to construct the background subspace via SVD, a constraint that is inherent to the spatiotemporal signal model.

The self-training strategy complements the A layer by extending supervision to unlabeled ultrasound data. The soft pseudo-labels preserve prediction fidelity rather than relying on hard binary decisions, reducing the risk of error propagation from uncertain teacher predictions. Despite the modest Dice gain of +0.0131 over the supervised UnrollNet, this improvement is consistent with the limited scale of the labeled dataset and suggests that unlabeled images provide additional supervision when grounded in the dynamics-based unrolling framework. On the extended test set, the SSL student showed higher median Dice (0.529 vs. 0.466) and IoU (0.360 vs. 0.304), and lower median trajectory error ($1.14^{\circ }$ vs. $1.84^{\circ }$) and tip error (1.25mm vs. 1.70mm) than the supervised UnrollNet, respectively (Table 4 and Figure 8).

The data efficiency ablation (Table 1 and Figure 3) shows that the dynamics-based inductive bias of the A layer partially compensates for annotation scarcity: at 50% labeled data, the proposed method achieves a Dice of 0.6965, approaching the supervised UnrollNet baseline of 0.7298 trained on the full labeled set without any unlabeled data.

Unlike MPL that continuously adapts the teacher via bi-level optimization on labeled data but relies entirely on data-driven pseudo-label feedback, the proposed method incorporates domain-specific dynamics constraints within the unrolling architecture, providing an additional supervisory signal that is independent of the labeled set size. This design proves particularly advantageous in our extremely label-scarce setting, where the limited labeled pool likely prevents MPL’s teacher updates from improving pseudo-label quality beyond the initial teacher performance, yielding MPL’s substantially lower Dice of 0.6426 despite achieving the lowest HD95 (8.02 pixels) and tip-position error (0.65mm) (Table 3). In contrast, Co-Training [17] with Swin U-Net [13] yields the lowest Dice, which can be attributed to the convergence difficulty of transformer-based architectures under data-scarce conditions. The Swin U-Net is unlikely to converge to a reliable representation with the limited labeled training data, and the resulting noisy pseudo-labels may actively degrade the U-Net component during co-training. It is worth mentioning that Co-Training may become competitive with the increasing label set, as transformer architectures are known to exploit their inductive bias more effectively with larger data volumes.

This work has several limitations. First, the SVD-based background subspace assumes a low-rank background estimated from consecutive frames, which may degrade under rapid tissue motion or non-stationary acoustic clutter and precludes single-frame inference; adaptive subspace tracking, such as recursive SVD or RPCA, could be incorporated in future work. Second, pseudo-label quality is bounded by the fixed supervised teacher, and an iterative teacher refinement such as the bi-level optimization in MPL [16] could be explored. Third, the network forward pass at N=5 required approximately 14.7ms per sample (batch size 1, NVIDIA RTX 3090), excluding the one-time background subspace estimation and data loading; real-time or in-procedure performance was not evaluated, and further optimization of the unrolling depth and the conjugate gradient solver would be required before such an evaluation. Finally, this work was restricted to a renal biopsy dataset from two institutions acquired with two ultrasound systems from the same vendor, with 181 labeled and 2653 unlabeled images and a small test set of 15 images, and the lower performance on the extended test set (Section 4.3) than on the original test set potentially indicates that the small test set does not fully represent the variability of clinical images. The generalizability across ultrasound systems, transducers, vendors, institutions, intervention types (hepatic, thyroid, and prostate), needle gauges, and insertion depths and angles therefore remains to be validated, and its strict analysis is beyond the scope of this work, though it needs future investigations. Extension to multi-center, multi-vendor and multi-organ data, potentially combined with domain adaptation, and prospective validation in clinical settings are required for practical use.

## 6. Conclusions

We successfully demonstrated the feasibility of a semi-supervised dynamics-based unrolling framework for ultrasound biopsy needle segmentation that integrates null-space projection with self-training to exploit unlabeled data under annotation-scarce clinical conditions. Compared with the supervised U-Net [9] and the supervised UnrollNet baselines, the proposed method achieves a Dice score of 0.7429±0.0489 and reduces the trajectory angle error to $0.66^{\circ }\pm 0.63^{\circ }$ and the tip-position error to 0.95mm±0.74mm, outperforming both supervised and semi-supervised baselines in Dice, IoU and trajectory angle error. It is expected that the proposed method may contribute to annotation-efficient deep learning for interventional ultrasound imaging, pending prospective, multi-center validation.

## Figures and Tables

**Table 1:** Data efficiency of the proposed method under varying proportions of labeled data. Bold indicates the best value in each metric. ↑ indicates that higher values are better, and ↓ indicates that lower values are better.

Label Proportion	Dice (↑)	IoU (↑)	HD95 (↓)	Traj. Error (↓)	Tip Error (↓)
10%	0.4865 ± 0.0964	0.3476 ± 0.2146	41.60 ± 30.72	1.48° ± 1.24°	1.91 mm ± 1.23 mm
20%	0.5849 ± 0.0788	0.4193 ± 0.1371	27.98 ± 26.69	1.65° ± 1.28°	1.69 mm ± 1.15 mm
30%	0.6331 ± 0.1043	0.4831 ± 0.1872	17.53 ± 17.79	1.72° ± 1.64°	**0.89 mm ± 0.60 mm**
50%	0.6965 ± 0.0461	0.5418 ± 0.1220	12.15 ± 9.93	1.28° ± 1.23°	0.91 mm ± 0.55 mm
100%	**0.7429 ± 0.0489**	**0.5967 ± 0.0917**	**10.69 ± 8.72**	**0.66° ± 0.63°**	0.95 mm ± 0.74 mm

**Table 2:** Ablation on the Analytic Model (A). Bold indicates the best value in each metric. ↑ indicates that higher values are better, and ↓ indicates that lower values are better.

Model	Dice (↑)	IoU (↑)	HD95 (↓)	Traj. Error (↓)	Tip Error (↓)
R-only (w/o A)	0.6184 ± 0.0913	0.4616 ± 0.1587	20.65 ± 21.00	1.65° ± 1.61°	1.32 mm ± 1.14 mm
Ours (R+A)	**0.7429 ± 0.0489**	**0.5967 ± 0.0917**	**10.69 ± 8.72**	**0.66° ± 0.63°**	**0.95 mm ± 0.74 mm**

**Table 3:** Quantitative comparison of segmentation and needle tracking performance. Bold indicates the best value in each metric, and italic indicates the method category (semi-supervised or supervised). ↑ indicates that higher values are better, and ↓ indicates that lower values are better.

Method	Dice (↑)	IoU (↑)	HD95 (↓)	Traj. Error (↓)	Tip Error (↓)
*Semi-supervised*					
Ours	**0.7429 ± 0.0489**	**0.5967 ± 0.0917**	10.69 ± 8.72	**0.66° ± 0.63°**	0.95 mm ± 0.74 mm
MPL	0.6426 ± 0.1140	0.4837 ± 0.1228	**8.02 ± 7.70**	1.55° ± 0.98°	**0.65 mm ± 0.61 mm**
Co-Training (U-Net)	0.6104 ± 0.0949	0.4462 ± 0.1016	19.26 ± 12.68	1.40° ± 1.02°	1.75 mm ± 1.45 mm
Co-Training (Swin)	0.5495 ± 0.1256	0.3892 ± 0.1197	16.36 ± 15.44	1.95° ± 1.24°	1.76 mm ± 1.87 mm
*Supervised*					
U-Net	0.5521 ± 0.1384	0.3938 ± 0.1311	30.84 ± 22.06	2.17° ± 3.28°	2.05 mm ± 1.80 mm
UnrollNet (sup.)	0.7298 ± 0.0518	0.5808 ± 0.1131	13.76 ± 12.91	1.20° ± 0.94°	1.08 mm ± 0.77 mm

**Table 4:** Quantitative comparison of the supervised UnrollNet (teacher) and the SSL student on the extended test set (N=176) (mean ± standard deviation). Bold indicates the best value in each metric. ↑ indicates that higher values are better, and ↓ indicates that lower values are better. Note that the SSL student shows higher Dice and IoU and lower trajectory and tip errors than the supervised UnrollNet, while HD95 is comparable between the two models.

Method	Dice (↑)	IoU (↑)	HD95 (↓)	Traj. Error (↓)	Tip Error (↓)
UnrollNet (sup.)	0.479 ± 0.188	0.336 ± 0.174	34.60 ± 23.78	2.31° ± 2.32°	2.28 mm ± 2.24 mm
UnrollNet (semi.)	**0.509 ± 0.206**	**0.366 ± 0.186**	**34.58 ± 27.42**	**1.51° ± 1.37°**	**1.49 mm ± 1.18 mm**

## Data Availability

The datasets used in this study were collected at Chonnam National University Hospital and Chosun University Hospital and are available upon reasonable request.

## Supplementary Materials

The following supporting information can be downloaded at https://www.mdpi.com/article/10.3390/diagnostics16203427/s1. Video S1: Needle segmentation inference results under normal imaging conditions; Video S2: Needle segmentation inference results under low visibility conditions; Video S3: Needle segmentation inference results under dark contrast conditions.
